# Role of Next-Generation Sequencing in Diagnosis of Familial Hypercholesterolemia in Serbia

**DOI:** 10.3390/diagnostics15101212

**Published:** 2025-05-12

**Authors:** Sandra Singh Lukac, Vladimir Gasic, Jovana Komazec, Ivana Grubisa, Ljiljana Popovic, Iva Rasulic, Sonja Pavlovic, Katarina Lalic

**Affiliations:** 1 Department for Lipid Disorders and Cardiovascular Complication in Diabetes, Clinic for Endocrinology, Diabetes and Metabolic Disease, University Clinical Centre of Serbia, 11000 Belgrade, Serbia; bastet224@yahoo.com (S.S.L.); liliana.popovich@gmail.com (L.P.); iva.kadic@yahoo.com (I.R.); katarina.s.lalic@gmail.com (K.L.); 2 Institute of Molecular Genetics and Genetic Engineering, University of Belgrade, 11042 Belgrade, Serbia; jovana.komazec@imgge.bg.ac.rs (J.K.); ivana.grubisa@imgge.bg.ac.rs (I.G.); sonja.pavlovic@imgge.bg.ac.rs (S.P.)

**Keywords:** familial hypercholesterolemia, NGS, *LDLR* gene, *APOB* gene

## Abstract

**Objectives:** Familial hypercholesterolemia (FH) is an autosomal dominant disorder of lipid metabolism characterized by high levels of low-density lipoprotein (LDL). This study aimed to identify variants in the *LDLR*, *APOB*, *PCSK9* and *LDLRAP1* genes and to identify the genotype–phenotype correlation in Serbian FH patients. **Method:** This study included a total of 101 patients suspected of having FH based on clinical criteria. Genetic analysis was performed by the next-generation sequencing (NGS) method. **Results:** An overall mutation detection rate of 43.6% was achieved. Thirteen distinct variants were detected in the *LDLR* gene (93.2%). The most frequently observed variant was c.858C>A p.(Ser286Arg), which was present in 26% of the LDLR-positive patients. Additional variants were detected in the *APOB* gene. No pathogenic variants were detected in the *PCSK9* or *LDLRAP1* genes. Comparing genetically FH-positive and FH-negative patients, statistical significance was observed in terms of age (*p* < 0.001), total cholesterol (TC) (*p* < 0.001), low-density-lipoprotein cholesterol (LDL-C) (*p* < 0.001) and triglyceridemia (*p* < 0.001). **Conclusions:** This study represents the first insight into the genetic basis of FH in Serbia. Taking into consideration that variants were detected in more than one gene and that the variants in the *LDLR* gene were distributed across nearly all exons, the FH diagnostics in Serbia ought to be based on NGS methodology.

## 1. Introduction

Familial hypercholesterolemia (FH) is a common inherited disorder of lipid metabolism characterized by a high level of low-density lipoprotein (LDL). Individuals with FH accumulate high cholesterol levels from birth, leading to prolonged exposure of blood vessels to elevated cholesterol and an increased risk of atherosclerosis and premature cardiovascular disease [1,2].

Familial hypercholesterolemia is usually an autosomal dominant disorder (heterozygous FH-HeFH). However, there is also a homozygous/compound heterozygous (HoFH) form. The presence of two altered alleles results in a more severe form of FH that usually appears in childhood. The disorder affects individuals from all ethnic groups, with a prevalence of HeFH from 1 to 313 and HoFH from 1 to 300,000 worldwide [3,4,5]. Despite its prevalence, FH remains significantly underdiagnosed and undertreated [6]. It is estimated that only 10% of affected individuals have received an accurate diagnosis [3].

The diagnosis of FH is typically based on established clinical diagnostic criteria, such as the Dutch Lipid Clinic Network Diagnostic Criteria (DLCN) or the Simon Broome Register Diagnostic Criteria [7,8,9]. These frameworks incorporate key factors, including personal or family history of premature atherosclerotic cardiovascular disease (ASCVD), elevated levels of LDL-C, and findings from physical examination [1].

Genetically, FH results from the presence of pathogenic variants in three key genes: *LDLR*, *APOB* and *PCSK9*, which are associated with the autosomal dominant form of FH [10]. The *LDLRAP1* gene is linked to the rare autosomal recessive form of the disease [10]. These genes play critical roles in regulating LDL cholesterol metabolism, and pathogenic variants in any of these genes can disrupt the normal clearance of LDL from the bloodstream [2,10,11].

The *LDLR* gene encodes the LDL receptor (LDLR), and pathogenic variants in this gene are the most common cause of FH, accounting for 80–85% of cases in affected individuals [10]. Over 2300 pathogenic variants in the *LDLR* gene have been associated with FH (Human Gene Mutation Database, HGMD, accessed July 2024). These include regulatory, missense, nonsense mutations, small indels, splice site changes and large deletions/insertions [12]. These alterations impair LDLR function by disrupting protein synthesis, causing retention in the endoplasmic reticulum, or affecting LDL binding, endocytosis and recycling [13].

The *APOB* gene encodes apolipoprotein B (apoB), a key component of LDL cholesterol. Pathogenic variants in the *APOB* gene lead to abnormal apoB, disrupting the binding of LDL to its receptor and reducing LDL clearance [14]. Around 400 pathogenic variants, mostly missense mutations (70%), have been reported in the *APOB* gene (HGMD, accessed July 2024). The *APOB* gene accounts for approximately 5 to 10% of all FH cases [10,12].

The *PCSK9* gene encodes proprotein convertase subtilisin/kexin type 9, a protein involved in the degradation of the LDLR. Pathogenic gain-of-function variants in *PCSK9* lead to increased receptor degradation and reduced LDL cholesterol clearance from the bloodstream [15]. These variants account for approximately 2 to 4% of all FH cases [16]. To date, around 100 pathogenic variants have been reported in the *PCSK9* gene, with missense mutations being the most common type (HGMD, accessed July 2024) [12].

Pathogenic variants in the *LDLRAP1* gene cause the extremely rare recessive form of FH, observed in less than 1% of FH patients. *LDLRAP1* interacts with the LDL receptor and aids in LDL-receptor-mediated endocytosis. Pathogenic variants in the *LDLRAP1* gene impair LDL clearance [10,17,18]. The HGMD database lists 35 variants in the *LDLRAP1* gene associated with FH (HGMD, accessed July 2024).

In Serbia, there are limited data on FH. It is estimated that up to 20% of FH patients have been identified, which translates to approximately 3000–7000 individuals. Notably, there are no genetically confirmed cases of FH in Serbia to date, which may be attributed to the limited availability of genetic testing for FH. Although FH can be diagnosed through clinical evaluation, genetic testing remains the gold standard for confirming the diagnosis and guiding appropriate management [19].

The aim of this study was to collect and present data on the frequency of pathogenic and likely pathogenic variants in the *LDLR*, *APOB*, *PCSK9* and *LDLRAP1* genes and identify the genotype–phenotype correlation in a Serbian cohort. We aimed to compare patients with and without FH-causing variants based on their clinical presentation indicative of FH. Our findings will contribute to the genetic diagnosis of FH in Serbia and facilitate cascade screening for early detection and treatment.

## 2. Materials and Methods

### 2.1. Study Population Sample

This study included patients suspected of having FH based on clinical criteria such as the Dutch Lipid Clinic Network diagnostic framework. A total of 101 patients (94 unrelated individuals and 7 family members) were recruited from the Unit for Lipid Disorders, Clinic for Endocrinology, Diabetes and Metabolic Diseases in Serbia between 2015 and 2023 by general practitioners, cardiology centers or other specialists. Inclusion criteria encompassed patients with elevated LDL-C levels (>5 mmol/L) without lipid-lowering therapy, a personal and/or family history of premature cardiovascular disease and/or physical findings suggestive of FH, such as the presence of cholesterol deposits like tendon xanthomas, xanthelasmas or corneal arcus. Patients were excluded from this study if they had medical conditions known to cause hyperlipidemia that were unrelated to FH, such as untreated hypothyroidism, nephrotic syndrome, cholestasis, hypopituitarism, severe obesity or chronic pancreatitis. Also, individuals who were using medications that could potentially alter lipid metabolism, including corticosteroids, immunosuppressive drugs, hormones, anabolic steroids or antipsychotics, were excluded. Demographic, clinical and biochemical data were collected to assess the prevalence, genetic spectrum and clinical implications of FH in patients enrolled in this study. Genetic testing for pathogenic variants in FH-associated genes was performed for all included participants.

This study was approved by the Research Ethics Committee of the Institute of Molecular Genetics and Genetic Engineering, University of Belgrade, number O-EO-059/2024. All patients have provided written informed consent to participate in this study.

### 2.2. Laboratory Analyses

Fasting serum lipid levels (total cholesterol (TC), high-density-lipoprotein cholesterol (HDL-C), and triglycerides (TGs)) were analyzed enzymatically using a commercial kit (Boehringer Mannheim GmbH Diagnostica, Germany). LDL-C was calculated using the standard Friedewald formula. Plasma glucose was measured using the glucose oxidase method (Beckman Instruments, Fullerton, CA, USA) [20].

### 2.3. FH Diagnosis

The diagnosis of FH was made using criteria from the DLCN score [21], which is based on patients’ clinical and family history data, physical examination, and LDL-C levels. Patients with a definite (DLCN score > 8), probable (DLCN score 6–8), possible (DLCN score 3–5), or unlikely FH diagnosis were included in this study.

### 2.4. Genetic Analysis

Patient’s genomic DNA was extracted from EDTA-treated whole blood samples using QIAamp Blood Mini kit (Qiagen, Dusseldorf, Germany). Library preparation was performed according to the manufacturer’s recommendations (Illumina Inc., San Diego, CA, USA). Genetic analysis was performed by the next-generation sequencing (NGS) method on the Illumina NextSeq 550DX platform (Illumina Inc., San Diego, CA, USA) using the TruSight One Sequencing Panel (Illumina, San Diego, CA, USA) for CES (clinical exome sequencing). A subset of genes relevant to FH (*LDLR* (NM_000527.4), *APOB* (NM_000384.2), *PCSK9* (NM_174936.4) and *LDLRAP1* (NM_015627.3)) was selected. Variants were prioritized using two software tools: Variant Interpreter (Illumina, San Diego, CA, USA) and VarSome (Saphetor, Lausanne, Switzerland). These variants were classified according to the recommendations of the ACMG (American College of Medical Genetics and Genomics) [22]. Variants found in patients were also searched in medical literature databases, such as ClinVAR (https://www.ncbi.nlm.nih.gov/clinvar/, accessed on 1 July 2024), HGMD (http://www.hgmd.cf.ac.uk/ac/index.php, accessed on 1 July 2024) and PubMed (https://www.ncbi.nlm.nih.gov/pubmed/, accessed on 1 July 2024) to ensure accuracy, determine clinical relevance, support diagnostic interpretation, and assess the novelty and clinical significance of the variants.

### 2.5. Statistical Analysis

Statistical analyses were performed in RStudio 2024.09.1 Build 394. The normality of the distribution of values in groups was investigated using the Shapiro–Wilk test. The comparison of means between different groups was performed using the Kruskal–Wallis test. Dunn’s test was used as a post hoc test. For examining the association of two categorical variables, the Chi-squared test was used. Spearman’s correlation was used to determine the dependencies of two continuous numerical variables. The Mann–Whitney U test was used to compare two independent samples of continuous values. The statistically significant *p* value was taken to be *p* < 0.001 to suggest very strong evidence.

## 3. Results

### 3.1. Study Population

A total of 101 patients were included in this study. The mean age of the patients was 55.1 years, and the standard deviation was 15.8 years. The mean concentration of TGs was 7.8 mmol/L, while the standard deviation was 2.8 mmol/L. The mean value of LDL-C was 5.4 mmol/L, and the standard deviation was 2.2 mmol/L. The mean concentration of HDL was 1.3 mmol/L, and the standard deviation was 0.34 mmol/L. The mean concentration of TGs was 1.9 mmol/L, while the standard deviation was 1.1 mmol/L. A total of 14.9% of patients had diabetes, and 32.7% had hypertension. Out of 101 patients, 11 had physical signs of FH.

### 3.2. Genetic Testing

Genetic testing was conducted on all 101 patients screened for the presence of pathogenic variants in genes associated with familial hypercholesterolemia (FH), specifically the *LDLR*, *APOB*, *PCSK9* and *LDLRAP1* genes. Pathogenic variants were identified in 44 out of the 101 patients, resulting in an overall detection rate of 43.6% using this method. Among the seven related individuals included in this study, originating from five different families, a causative variant was identified in three families (c.81C>G p.(Cys27Trp) was found in two families, and c.1646G>A p.(Gly549Asp) in one family), while in the remaining two families, no causative variant could be detected. The DLCN classification of patients suspected of having FH and the genetic confirmation rates are summarized in Table 1. Pathogenic variants were detected in 83.9% of patients classified as definitive familial hypercholesterolemia (DFH) and in 20.0% of those classified as probable familial hypercholesterolemia. In contrast, 28.1% and 25.0% of genetically confirmed patients were classified as possible and unlikely familial hypercholesterolemia, respectively (Table 1).

By comparing genetically FH-positive and FH-negative patients, statistical significance was observed in terms of age (*p* < 0.001), total cholesterol (TC) (*p* < 0.001), low-density-lipoprotein cholesterol (LDL-C) (*p* < 0.001) and triglyceridemia (*p* < 0.001). No other statistically significant associations were found.

The lipid profile in genetically confirmed FH patients shows a statistically significant elevation in LDL-C and total cholesterol, consistent with the pathophysiology of FH. In our cohort, patients carrying the heterozygous variant c.858C>A p.(Ser286Arg) had the lowest LDL-C levels, although this difference was not statistically significant, and the levels approached the average LDL-C value seen in the overall group. Normal HDL-C levels and mildly elevated triglycerides were also observed (Table 2).

In this study, we found that 93.2% (41/44) of patients had a pathogenic variant in the *LDLR* gene. Thirteen distinct variants were detected, and all but two were heterozygous. Only one patient had a homozygous variant, and another had two heterozygous variants in the *LDLR* gene. The most frequently observed variant was c.858C>A p.(Ser286Arg), which was present in 26% of the LDLR-positive patients. Notably, this variant was also present in the only patient with a homozygous variant and in one with two heterozygous variants. The second most common variant was c.81C>G p.(Cys27Trp), which was found in 17.1% of the LDLR-positive patients (Table 3).

**Figure 1:**
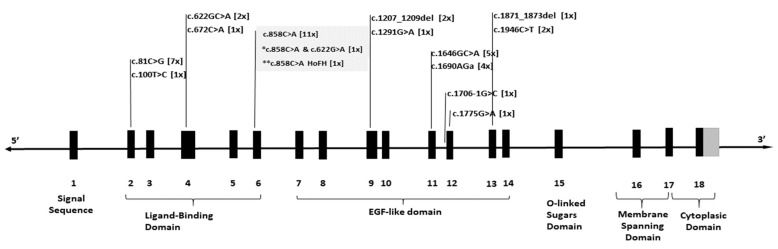
Schematic representation of the LDLR gene, showing exons (bold bars) and introns (lines). The figure highlights detected variants and their positions within the gene, with the number of patients carrying each variant indicated in square brackets. * denotes a patient with compound heterozygous varinats c.858C>A and c.622G>A; ** denotes a patient with homozygous variant c.858C>A.

Variants detected in the *LDLR* gene were distributed across nearly all exons. Specifically, five variants mapped to exons 2–6, which encode the ligand-binding domain, whereas eight variants localized to exons 7–13, encompassing the EGF-like domain (Figure 1).

In the *APOB* gene, we identified one variant, the most frequent variant in the *APOB* gene, namely c.10580G>A p.(Arg3527Gln), in three unrelated patients, representing 6.8% of all patients in the cohort.

No pathogenic variants were detected in the *PCSK9* or *LDLRAP1* genes.

## 4. Discussion

The clinical presentation of familial hypercholesterolemia (FH) is highly variable, with significant differences in severity, the presence of tendon xanthomas, xanthelasmas, age of onset, and the risk of developing plaques in coronary, cerebral or peripheral vasculature. These plaques can lead to coronary artery disease (CAD), stoke and limb ischemia [23,24]. Additionally, the physical manifestations of FH typically develop later in life, leading to potential oversights in younger patients [25]. For this reason, relying solely on clinical criteria for diagnosis may not be reliable and could potentially miss a significant number of FH patients, particularly in cases where family history is unavailable or unknown. Therefore, additional diagnostic methods, such as genetic testing, may be necessary to accurately identify FH patients.

In this study, patients with a clinical diagnosis of FH underwent genetic analysis using the next-generation sequencing method. A total of 44 individuals were found to have FH-related variants, all of which had been previously reported and classified as pathogenic according to the ACMG criteria [22]. However, modified ACMG classification criteria for the *LDLR* gene have been established. Therefore, certain variants could be re-classified according to those guidelines [26].

Variants in the *LDLR* gene identified in this study were found scattered throughout the entire length of the *LDLR* gene, with no apparent clustering. This is consistent with what had been seen in previous studies on familial hypercholesterolemia in different populations [27,28,29,30,31,32,33]. All variants in our study were found in the ligand-binding and epidermal growth factor-like domain, which is consistent with existing current knowledge on the distribution of most variants in the *LDLR* gene [34]. The majority of variants in the *LDLR* gene are found in exons that encode functionally critical domains. In particular, pathogenic variants in the ligand-binding domain can impair the receptor’s ability to bind LDL particles, leading to elevated LDL cholesterol levels [35]. Additionally, the epidermal growth factor domain is crucial for the proper folding, trafficking and functioning of the LDL receptor. Pathogenic variants within this domain can disrupt these processes, resulting in impaired LDL receptor function and leading to an increase in LDL cholesterol levels [34].

Among the variants found in our study, one particular variant, c.858C>A p.(Ser286Arg), which causes a change from serine to arginine at position 286 in the protein, was more common than the other variants we observed. This variant was present in about one-third of the patients who tested positive for the *LDLR* gene.

The c.858C>A p.(Ser286Arg) variant appears to be quite rare among various populations or not observed [27,29,31,32,36]. However, the Greek population presents an exception, where it is the second most common cause of familial hypercholesterolemia and is associated with a milder phenotype [30,37]. Consistent with the Greek study, our results show that this variant is the most frequent in our cohort and that patients carrying it have the lowest LDL-C levels compared to those with other *LDLR* or *APOB* gene variants, indicative of a milder phenotype. Patients carrying the heterozygous *LDLR* gene variant c.858C>A p.(Ser286Arg) presented with a mean age of 49 or 50 years, suggesting that they come to clinical attention later in life than other LDL-positive patients. This indicates that these individuals may be overlooked or misdiagnosed with other conditions, potentially delaying the diagnosis of familial hypercholesterolemia. In contrast, patients with other variants in the *LDLR* gene (not c.858C>A) exhibited higher levels of LDL-C and total cholesterol (TC), as well as an earlier age of presentation.

In our study, the c.858C>A p.(Ser286Arg) variant was found in a homozygous state in one patient and co-occurred with the c.622G>A p.(Glu208Lys) variant in another patient. The phenotype of HoFH is more severe, characterized by very high LDL-C levels, physical signs of cholesterol deposits and a premature CAD usually before 20 years of age [11,38,39]. Over 95% of patients with HoFH carry two different variants in the *LDLR* gene (compound heterozygous), while the remaining cases have identical variants in both alleles (true homozygous) or variants in different FH-related genes [11,40]. The patient carrying the homozygous c.858C>A p.(Ser286Arg) variant presented with xanthomas and a history of coronary heart disease, with family members similarly affected by high LDL-C levels or heart attacks. The patient had TC and LDL-C levels of 8.72 mmol/L and 7.17 mmol/L, respectively. To our knowledge, a homozygous patient carrying this variant has not been previously reported in the literature.

On the other hand, the patient with the compound genotype exhibited a more severe clinical presentation, having much higher TC and LDL-C levels, including xanthomas, hypertension and diabetes. In the absence of samples for segregation analysis and relying on clinical characteristics, we strongly suspect that the c.622G>A p.(Glu208Lys) variant contributes to a compound heterozygous genotype. Patients with compound heterozygous variants tend to exhibit a more severe phenotype than those with simple heterozygous variants, closely resembling the HoFH phenotype [41]. To our knowledge, a compound heterozygous patient carrying these two variants has not been previously reported in the literature. However, the c.858C>A p.(Ser286Arg) variant has been reported in a compound state with another variant, c.517T>C p.(Cys173Arg), where both variants were shown to reduce LDLR activity [42]. Functional studies have shown that, independently, both variants—c.858C>A p.(Ser286Arg) and c.622G>A p.(Glu208Lys)—cause the LDLR to retain only 5–15% of its normal activity [42]. Furthermore, it has been demonstrated that the glutamic acid at position 208 is involved in the formation of the binding site for very-low-density lipoprotein (VLDL) particles, specifically for apolipoprotein E, and its substitution with lysine (Lys) at this position reduces the receptor’s affinity for VLDL particles [43]. However, the genotype–phenotype correlation in our patient, homozygous for c.858C>A p.(Ser286Arg) variant, indicates that the effect of this variant could be classified as mild.

Genetic analysis of the *APOB* gene in this cohort revealed the presence of only one variant, the c.10580G>A p.(Arg3527Gln). This variant, also known as p.(Arg3500Gln) or R3500Q in earlier literature, is a well-known pathogenic variant, first described over 30 years ago and associated with familial hypercholesterolemia [44]. The variant creates a substitution of glutamine for arginine at a codon 3527 p.(Arg3527Gln), leading to reduced affinity for the LDL receptor and impaired uptake of LDL into the cell [45,46]. Carriers of the variant show elevated levels of LDL-C [47], which are also seen among patients in this cohort.

This variant is the most commonly reported in the *APOB* gene and has been found to have varying frequencies across different studies, ranging from 0.02% to as high as 68% when patients clinically characterized as DFH were included and specifically screened for *APOB* pathogenic variants [48]. The 7% frequency observed in our study aligns with most studies, where *APOB* pathogenic variants typically occur in less than 10% of FH cases [27,28,29].

In our study, a genetic cause for FH was not identified in a small portion of patients clinically diagnosed with DFH (less than 15%) and a larger proportion of those diagnosed with PFH (80%). These findings align with a previous study that has also reported the absence of a genetic cause in a similar proportion of FH cases [49]. Several factors may contribute to this finding. One key factor is the limitations of the testing method used, which does not detect copy number variations (CNVs), found in around 10% of FH patients, or deep intronic variants [50]. This issue could potentially be addressed with whole-genome sequencing (WGS), which provides a more comprehensive genetic analysis.

It is also possible that some FH-related genes have not yet been identified, indicating that further genetic factors contributing to the disease remain undiscovered. Moreover, in patients who test genetically negative for FH, the most plausible explanation for their clinical diagnosis of FH is a polygenic basis [51]. Common genetic variants in the *LDLR* and *APOB* genes have been shown to have a small cumulative effect on increasing LDL-C levels [24,52]. The lack of statistically significant associations between the genes in which pathogenic and likely pathogenic variants have been found could be attributed to a lack of understanding of the regulatory mechanisms of cholesterol uptake. Lastly, the possibility of false-positive clinical diagnoses should also be considered.

Conversely, we observed that seven patients classified as “unlikely” to have FH by the DLCN criteria were found to carry a causative genetic variant. This underscores the limitations of clinical scoring systems. Amor-Salamanca et al. reported that 44% of genetically confirmed FH cases were missed by DLCN, and Tada et al. similarly found the DLCN criteria to be less sensitive than the Japan Atherosclerosis Society (JAS) criteria [53,54]. These findings support the use of genetic testing alongside clinical assessment to enhance diagnostic accuracy.

The mutation detection rate in this study was achieved using an NGS approach. No other methodology could be so efficient. We have used an exome sequencing approach, but whole-genome sequencing would be even more useful. It would enable not only the detection of all variants in known genomic regions associated with FH, but also the discovery of new genes that could be disease-causing or disease-associated in FH patients.

## 5. Conclusions

While there is currently no cure for familial hypercholesterolemia, a genetic diagnosis can be advantageous to patients in several ways. It allows personalized management of their condition through appropriate therapy, such as a specific molecular targeted therapy (available for patients who are carriers of variants in the *PCSK9* gene). Knowledge of the genetic basis of FH also enables early testing and treatment of relatives before severe symptoms develop and facilitates control of high cholesterol levels and lifestyle modifications to prevent or reduce the risk of atherosclerosis and coronary artery disease. Given the frequency of the c.858C>A p.(Ser286Arg) variant in our cohort and its association with a milder phenotype, there is a risk of overlooking patients who carry this variant. A genetic diagnosis for these patients would not only confirm the clinical diagnosis but also facilitate early intervention, as well as testing for family members. This could significantly enhance both the management and prognosis of the disorder in those patients.

This study represents the first insight into the genetic basis of FH in Serbia. It confirmed that the NGS approach is a method of choice for FH genetic diagnosis. It will be used in the future genetic screening of all patients suspected of FH.

## Figures and Tables

**Table 1:** DLCN classification of familial hypercholesterolemia patients and genetic confirmation rates.

Diagnosis (DLCN Criteria)	No. of FH Patients (DLCN Classification)	No. of Genetically Confirmed	% of Genetically Confirmed
Definite	31	26	83.9%
Probable	10	2	20.0%
Possible	32	9	28.1%
Unlikely	28	7	25.0%

**Table 2:** Clinical features of FH patients: comparison between FH-negative, FH-positive, and subgroups based on genetic findings.

Clinical Parameters Being Measured (Mean ± SD)	All Patients (*n* = 101)	Genetically FH-Negative Patients (*n* = 57)	Genetically FH-Positive Patients (*n* = 44)	LDLR-Positive Patients (*n* = 41)	LDLR-Positive Patients Without the c.858C>A p.(Ser286Arg) Variant (*n* = 28)	LDLR-Positive Patients with the c.858C>A p.(Ser286Arg) (*n* = 13)	LDLR-Positive Patients with the c.858C>A p.(Ser286Arg) Variant Excluding HoFH/CoFH (*n* = 11)	APOB-Positive Patients (*n* = 3)
Age (years)	55.1 ± 15.8	60.8 ± 14.9 *	48.0 ±14.1 *	48.5 ± 14.3	47.8 ±16.3	50.0 ± 9.26	49.1 ± 8.98	40.0 ± 10.6
Gender (F/M)	57/44	32/25	25/19	17/24	15/13	9/4	8/3	1/2
TC (mmol/L)	7.8 ± 2.8	6.7 ± 2.0 *	9.3 ± 3.1 *	9.3 ± 3.2	9.7 ± 3.5	8.5 ± 2.4	8.04 ± 1.94	8.30 ± 0.96
LDL-C (mmol/L)	5.4 ± 2.2	4.4 ± 1.7 *	6.7 ± 2.3 *	6.7 ± 2.3	6.9 ± 2.4	6.2 ± 2.2	5.69 ± 1.94	6.50 ± 1.1
HDL-C (mmol/L)	1.3 ± 0.34	1.3 ± 0.28	1.4 ± 0.4	1.4 ± 0.41	1.4 ± 0.43	1.4 ± 0.39	1.49 ± 0.38	1.2 ± 0.11
TGs (mmol/L)	1.9 ± 1.1	2.1 ± 0.98 *	1.6 ± 1.2 *	1.6 ± 1.2	1.7 ± 1.4	1.5 ± 0.75	1.57 ± 0.8	1.4 ± 0.21
Glycemia (mmol/L)	5.6 ± 1.7	5.7 ± 1.3	5.5 ± 2.1	5.6 ± 2.2	5.4 ± 2.0	5.9 ± 2.6	5.34 ± 0.76	4.90 ± 0.36
BMI (kg/m^2^)	25 ± 3.7	26 ± 3.1	25.0 ± 4.4	25.0 ± 4.5	24.0 ± 4.3	27 ± 4.90	26.2 ± 5.13	24.0 ± 1.20

TC—total cholesterol; LDL-C—low-density-lipoprotein cholesterol; HDL-C—high-density-lipoprotein cholesterol; TGs—triglycerides; HoFH—homozygous familial hypercholesterolemia; CoFH—compound heterozygous familial hypercholesterolemia; * *p* value—indicates statistical significance at *p* < 0.001 for each comparison (between FH-negative and FH-positive patients).

**Table 3:** Spectrum of pathogenic variants in the *LDLR* and *APOB* genes in the Serbian cohort.

No. P.	Exon Number	Gene	Zygosity	Variant	Variant Type	ACMG Classification	Previous Description
7	2	LDLR	het	c.81C>G p.(Cys27Trp)	ms	P	ClinVar 226304
1	2	LDLR	het	c.100T>C p.(Cys34Arg)	ms	P	ClinVar 251018, PMID: 33231818
2	4	LDLR	het	c.622G>A p.(Glu208Lys)	ms	P	ClinVar 251328
1	4	LDLR	het	c.672C>A p.(Asp224Glu)	ms	P	ClinVar 375790
11	6	LDLR	het	c.858C>A p.(Ser286Arg)	ms	P	ClinVar 251488
1	6	LDLR	hom	c.858C>A p.(Ser286Arg)	ms	P	ClinVar 251488
1	6	LDLR	Comp. het	c.858C>A p.(Ser286Arg)	ms	P	ClinVar 251488
	4			c.622G>A p.(Glu208Lys)	ms	P	ClinVar 251328
2	9	LDLR	het	c.1207_1209del p.(Phe403del)	ind	P	ClinVar 251731
1	9	LDLR	het	c.1291G>A p.(Ala431Thr)	ms	P	ClinVar 3695
5	11	LDLR	het	c.1646G>A p.(Gly549Asp)	ms	P	ClinVar 3698
4	11	LDLR	het	c.1690A>G p.(Asn564Asp)	ms	P	ClinVar 251973
1	intron 11	LDLR	het	c.1706-1G>A	ss	P	ClinVar 251992
1	12	LDLR	het	c.1775G>A p.(Gly592Glu)	ms	P	ClinVar 161271
1	13	LDLR	het	c.1871_1873del p.(Ile624del)	ifd	P	ClinVar 250297
2	13	LDLR	het	c.1946C>T p.(Pro649Leu)	ms	P	ClinVar 252122
3	26	APOB	het	c.10580G>A p.(Arg3527Gln)	ms	P	ClinVar 17890

No. P.—number of patients carrying a variant; GeneBank accession numbers for genes: *LDLR*: NM_000527.4, *APOB*: NM_000384.2. ACMG classification: P—pathogenic; ms—missense; ss—splice site; ifd—in-frame deletion; comp. het—compound heterozygote.

## Data Availability

The data are available only upon request, due to a non-disclosure agreement provided by the institution.

## Abbreviations

The following abbreviations are used in this manuscript:

ASCVD	Atherosclerotic cardiovascular disease
APOB	Apolipoprotein B
CAD	Coronary artery disease
DFH	Definitive familial hypercholesterolemia
DLCN	Dutch Lipid Clinic Network Diagnostic Criteria
FH	Familial hypercholesterolemia
HGMD	Human Gene Mutation Database
HeFH	Heterozygous familial hypercholesterolemia
HoFH	Homozygous form of familial hypercholesterolemia
JAS	Japan Atherosclerosis Society
LDL	Low-density lipoprotein
LDLR	Low-density lipoprotein receptor
LDLRAP1	Low-density lipoprotein receptor adapter protein 1
NGS	Next-generation sequencing
PCSK9	Proprotein convertase subtilisin/kexin type 9
PFH	Probable familial hypercholesterolemia
VLDL	Very-low-density lipoprotein
